# Development of the intestinal microbiome in cystic fibrosis in early life

**DOI:** 10.1128/msphere.00046-23

**Published:** 2023-07-05

**Authors:** Courtney E. Price, Thomas H. Hampton, Rebecca A. Valls, Kaitlyn E. Barrack, George A. O’Toole, Juliette C. Madan, Modupe O. Coker

**Affiliations:** 1 Department of Microbiology and Immunology, Geisel School of Medicine at Dartmouth, Hanover, New Hampshire, USA; 2 Department of Pediatrics, Children’s Hospital at Dartmouth, Dartmouth Health, Lebanon, New Hampshire, USA; 3 Department of Psychiatry, Children’s Hospital at Dartmouth, Dartmouth Health, Lebanon, New Hampshire, USA; 4 Department of Epidemiology, Geisel School of Medicine at Dartmouth, Hanover, New Hampshire, USA; 5 Department of Quantitative Biomedical Data Science, Geisel School of Medicine at Dartmouth, Hanover, New Hampshire, USA; 6 Department of Oral Biology, Rutgers School of Dental Medicine, Newark, New Jersey, USA; University of Michigan-Ann Arbor, Ann Arbor, Michigan, USA

**Keywords:** cystic fibrosis, gut, microbiome, Crohn’s disease

## Abstract

Cystic fibrosis (CF) is a heritable disease that causes altered physiology at mucosal sites; these changes result in chronic infections in the lung, significant gastrointestinal complications as well as dysbiosis of the gut microbiome, although the latter has been less well explored. Here, we describe the longitudinal development of the gut microbiome in a cohort of children with CF (cwCF) from birth through early childhood (0–4 years of age) using 16S rRNA gene amplicon sequencing of stool samples as a surrogate for the gut microbiota. Similar to healthy populations, alpha diversity of the gut microbiome increases significantly with age, but diversity plateaus at ~2 years of age for this CF cohort. Several taxa that have been associated with dysbiosis in CF change with age toward a more healthy-like composition; notable exceptions include *Akkermansia*, which decreases with age, and *Blautia*, which increases with age. We also examined the relative abundance and prevalence of nine taxa associated with CF lung disease, several of which persist across early life, highlighting the possibility of the lung being seeded directly from the gut early in life. Finally, we applied the Crohn’s Dysbiosis Index to each sample, and found that high Crohn’s-associated dysbiosis early in life (<2 years) was associated with significantly lower *Bacteroides* in samples collected from 2 to 4 years of age. Together, these data comprise an observational study that describes the longitudinal development of the CF-associated gut microbiota and suggest that early markers associated with inflammatory bowel disease may shape the later gut microbiota of cwCF.

**IMPORTANCE:**

Cystic fibrosis is a heritable disease that disrupts ion transport at mucosal surfaces, causing a buildup of mucus and dysregulation of microbial communities in both the lungs and the intestines. Persons with CF are known to have dysbiotic gut microbial communities, but the development of these communities over time beginning at birth has not been thoroughly studied. Here, we describe an observation study following the development of the gut microbiome of cwCF throughout the first 4 years of life, during the critical window of both gut microbiome and immune development. Our findings indicate the possibility of the gut microbiota as a reservoir of airway pathogens and a surprisingly early indication of a microbiota associated with inflammatory bowel disease.

## INTRODUCTION

Cystic fibrosis (CF) is a heritable disease caused by mutations in the cystic fibrosis transmembrane conductance regulator (CFTR). Loss of CFTR function leads to altered secretion of chloride and bicarbonate and accumulation of abnormally thick mucus in both the lungs and the intestinal tract (1, 2). Loss of CFTR function also alters bile acid production and diminishes secretion of pancreatic enzymes. Persons with CF (pwCF) can experience intestinal blockages at birth (meconium ileus), bacterial overgrowth in the small bowel, inflammation, dysmotility, and often struggle with sufficient weight gain in early childhood (3).

More recently, several studies have documented alterations in the gut microbiome for pwCF, as well as associations between the gut microbiome and health outcomes (4 - 10). Alterations in the gut microbiome are associated with, and may contribute to, important clinical outcomes, including increased inflammation, maldigestion, malabsorption, intestinal lesions, and poor linear growth (8, 11 - 14). Emerging evidence indicates that the gut–lung axis, wherein the health of the intestinal microbiome affects distal organ health, is an important determinant of lung health outcomes for pwCF (5 - 7), likely via immune programming. Studies have shown that weight gain is associated with better pulmonary outcomes and that in early life the gut microbiome is a better predictor of lung health outcomes than the respiratory microbiome (15). Microbiome alterations are driven by CFTR mutations and are, therefore, inherent to CF (10, 16) but are also likely influenced by CF clinical manifestations, such as pancreatic sufficiency and CFTR genotype (17 - 19). CF gut microbiome health can also be influenced by external exposures, such as breastfeeding or delivery mode, and by treatment with CF-specific drug regimens, probiotics, or antibiotics (5, 20 - 23). However, studies comparing microbiome development in children with and without CF point to the decreased influence of typical exposures, such as breastfeeding and antibiotic exposures, on the microbiome composition, highlighting the importance of CFTR mutations and the associated micro- and macroenvironments in CF (4, 9).

For pwCF, gut microbiome dysbiosis begins in early childhood and continues into adolescence and adulthood (17, 23). Infants and children with CF (cwCF) have been shown to have lower alpha diversity (4, 9, 11, 17, 24) as well as delayed microbiome maturation relative to healthy cohorts (8). Additionally, cwCF have many of the same alterations noted in adults, including increased *Veillonella* and *Escherichia coli*, and lower *Bacteroides, Faecalibacterium,* and *Akkermansia*. However, dysbiosis is most pronounced in early infancy, and some differences between CF and healthy cohorts are age dependent (8, 17). Furthermore, an age-based analysis revealed that CF intestinal microbiomes appeared more immature than microbiomes of healthy children, which could have implications for immune development (8).

The early window of gut microbiome maturation (0 to ~4 years) is particularly important because development of the immune system occurs during this same time period, and a healthy gut microbiome is essential for proper immune training (25 - 27). This early developmental window has only been fully analyzed in two CF studies (9, 28) and partially covered by several others (4, 8, 11, 16, 17, 24, 29, 30). CF microbiome studies to date have primarily focused on either early microbiomes (<1 year) or adult microbiomes, and a few small studies have sampled at larger intervals throughout childhood (8, 9, 28). Here, we characterize fecal microbiomes from a cohort of 39 children across the first 4 years of life, with samples taken frequently throughout this important developmental period.

## MATERIALS AND METHODS

### Sample collection and sequencing

Stool samples were collected by parents of cwCF and initially stored in a home freezer. Once samples arrived in the lab, they were stored at −80°C and later processed with Zymo fecal DNA miniprep kit (Cat #D6010). Paired-end reads were generated with 2 × 250 Illumina Miseq amplicon sequencing of the V4–V5 hypervariable region of the 16S rRNA gene (Woods Hole Marine Biological Laboratory). Raw data have been uploaded to the NCBI sequence read archive under accession number PRJNA170783 (SRP014429) and BioProject ID 170783.

### Processing of rRNA-encoding gene amplicons

Primer sequences were removed by CUTADAPT (version 1.18). All subsequent preprocessing steps were performed in R version 3.6.0. Code is available at https://github.com/GeiselBiofilm. A total of 39,577,426 raw paired-end reads were filtered and trimmed with DADA2 version 1.14.1. Reads were then denoised, merged, and chimeras removed. The final counts were 30,765,091 total reads, 109,096 mean reads per sample, and 2,726 unique amplicon sequence variants (ASVs). Taxonomy was assigned with DADA2 and the Silva version 138.1 training set. ASV tables and sample information are available in the supplemental tables (Table S1 and S2).

### Analysis of 16S rRNA-encoding gene amplicons

All downstream analysis and visualization were performed in R (version 4.0.2). Phyloseq (version 1.32.0) and ggplot2 (version 3.3.2) were used for data handling and visualization unless otherwise noted (31). Reads per sample were graphed and filtered to include only samples with >10,000 reads. One sample was removed because of low read count (<10^4^ reads), and the remaining 281 samples were further analyzed. DESeq2 (version 1.28.1) was used to call significantly different abundances as described by McMurdie and Holmes (32, 33). Linear mixed models were used for statistical regression analysis (nlme version 3.1.151). For each sample, beta diversity was calculated by Bray–Curtis distance, and multidimensional scaling ordination was performed. Significant differences in beta diversity were tested by permutational analysis of variance (PERMANOVA) (vegan version 2.5.7).

### Crohn’s Dysbiosis Index

The Crohn’s Dysbiosis Index (CDI) was applied to all samples as originally described by Gevers et al. and first applied to CF by Enaud et al. (12, 34). Briefly, counts of taxa associated with both positive and negative relative abundance changes in Crohn’s disease (CD) were summed for each sample. The log_10_ ratio of the (microbes increased in CD)/(microbes decreased in CD) was then calculated for each sample and associated with the relevant subject data.

## RESULTS AND DISCUSSION

### Subject population and metadata

We analyzed data from a cohort of 39 children with CF. Stool samples were collected longitudinally from 13 days up to 48 months of age (Table 1). A total of 281 stool samples were analyzed. Each subject had between 1 and 15 samples collected, with a median of 8 samples collected per subject. Data recorded from each cwCF included sex, genotype, pancreatic sufficiency, delivery mode, preterm birth status, and whether the child was ever breastfed. A summary of this information is provided in Table 1. Metadata for each sample was also collected, including recent and long-term antibiotic exposure information and whether the cwCF had a recent oropharyngeal (OP) swab with a positive culture for the common CF lung pathogens *Pseudomonas aeruginosa* or *Staphylococcus aureus* (Table S1).

**TABLE 1:** Subject and sample metadata

Subject data		No. of subjects	No. of samples	% of subjects	% of samples
Pancreatic sufficiency	Sufficient	5	33	12.8	11.7
	Insufficient	33	243	84.6	86.5
	Not reported	1	5	2.6	1.8
Genotype	dF508/dF508	20	157	51.3	55.9
	dF508/other	14	93	35.9	33.1
	Rare/rare	3	23	8.1	8.4
	Not reported	2	8	5.4	2.9
Sex	Female	20	158	51.3	56.2
	Male	17	115	43.6	40.9
	Not reported	2	8	5.1	2.8
Breastfed ever	Yes	20	150	51.3	53.4
	No	18	126	46.2	44.8
	Not reported	1	5	2.6	1.8
Delivery mode	Vaginal	23	167	59.0	59.4
	C-section	14	106	35.9	37.7
	Not reported	2	8	5.1	2.8
Preterm	Full	29	233	74.4	82.9
	Pre	6	32	15.4	11.4
	Not reported	4	16	10.3	5.7

### Age is the strongest influence on early cystic fibrosis microbiome alpha and beta diversity

In healthy cohorts, age is the dominant factor shaping the structure of the microbiome, but early life exposures also impact microbiome structure (35). In this study, we wanted to determine whether the intestinal microbiome of cwCF exhibits known maturation patterns, including increases in microbial diversity over time and age-dependent changes in microbial composition. Additionally, we wanted to test whether early life exposures impact microbiome development patterns in the short and long term. We used a mixed linear model with subject as the random effect to test whether Shannon Diversity Index (SDI), an alpha diversity measure encompassing both microbial richness and evenness, was significantly correlated with gender, pancreatic sufficiency, premature birth, delivery mode, breastfeeding, age, recent antibiotic exposure, and recent OP swab positive for *P. aeruginosa* or *S. aureus*.

**Fig 1:**
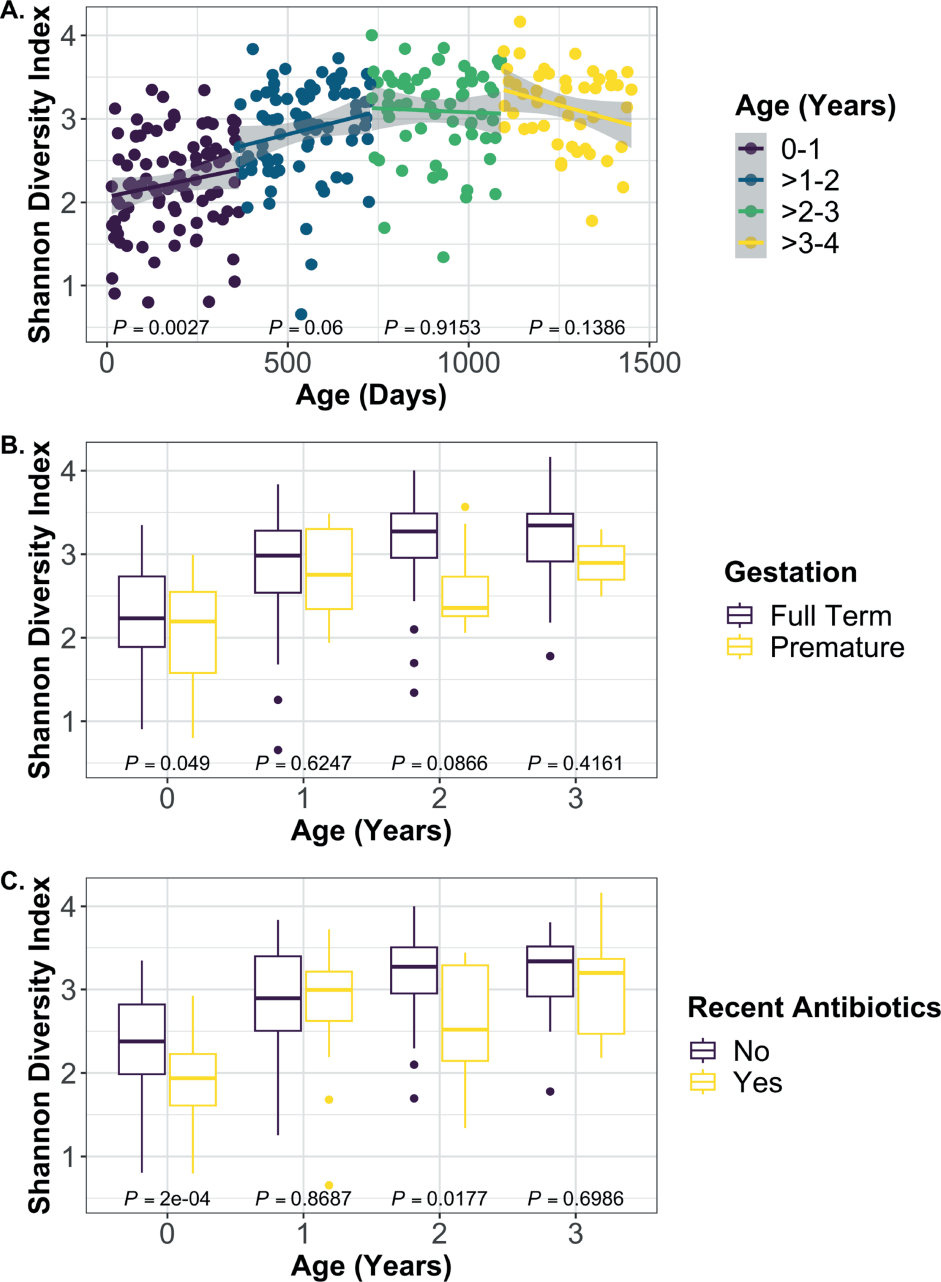
Alpha diversity is significantly associated with age, gestation, and recent antibiotic exposure. A linear mixed effects model from the R package nlme was used to test whether SDI changed significantly with age, gestation, or recent antibiotic exposure when all samples were included in the model. Subject was set as the random variable to control for multiple sampling. SDI was significantly positively correlated with age in days (*P* = 4.14e-19), full-term gestation (*P* = 0.0236), and absence of antibiotic exposure within the previous 60 days (*P* = 0.00995). (**A**) For each sample, subject age in days is graphed versus the Shannon Diversity Index. A linear model was used to visualize the increase in diversity over the first 2 years of life and the plateau of SDI above 2 years of age. (**Band C**) SDI is graphed for each sample divided by 1 year age bins and (B) gestation or (C) recent antibiotic exposure within the last 60 days. A linear mixed effects model was used to test for significant associations within each 1 year age bin, and *P*-values are reported on the graphs.

Total antibiotic exposure (defined as all antibiotic treatments over the period where samples were collected for each subject) prior to sample collection was not included in the model due to a significant interaction with age (*P* = 0.0107). Insignificant variables were then sequentially removed to avoid overfitting of the model, leaving age in days, gestation (i.e., preterm versus full term), and recent antibiotic exposure, defined as antibiotics taken in the previous 60 days, as significant factors influencing SDI (Fig. 1; age *P* = 4.14e-19; gestation *P* = 0.0236; recent antibiotics *P* = 0.00995).

Age and SDI were positively correlated (Fig. 1A), with a visible plateau in SDI beginning at approximately 2 years of age (Fig. S1A). When the same linear model was repeated on samples binned by subject age, <2 years or 2–4 years, SDI significantly increased with age over the first 2 years of life (*P* = 2.58e-13) but not from 2 to 4 (*P* = 0.960) years of age (Fig. S1B and C; Table S1). Premature birth was associated with significantly lower SDI than full-term birth over the first 4 years of life ( Fig. S1D and E). Interestingly, when this analysis was performed on samples grouped in 1 year age bins, cwCF born prematurely had significantly different SDI <1 year of age, but SDI trended lower in the premature group up to 4 years of age, suggesting that premature birth may have long-term impacts on the maturation of the gut microbiota in cwCF (Fig. 1B).

Recent antibiotic exposure was determined by whether the cwCF had non-topical antibiotic treatment up to 60 days prior to sample collection, a cutoff previously used in a study of cwCF (9). Recent antibiotic exposure was significantly associated with a less diverse gut microbiota during the first year of life and from 2 to 3 years of age (Fig. 1C; Fig. S1F and G). We note that it is difficult to attribute a role for antibiotic treatment regimen or a specific antibiotic on microbial composition, given the relatively small cohort size and the complexity of antibiotic exposures over the first year of life (Fig. S2A).

**Fig 2:**
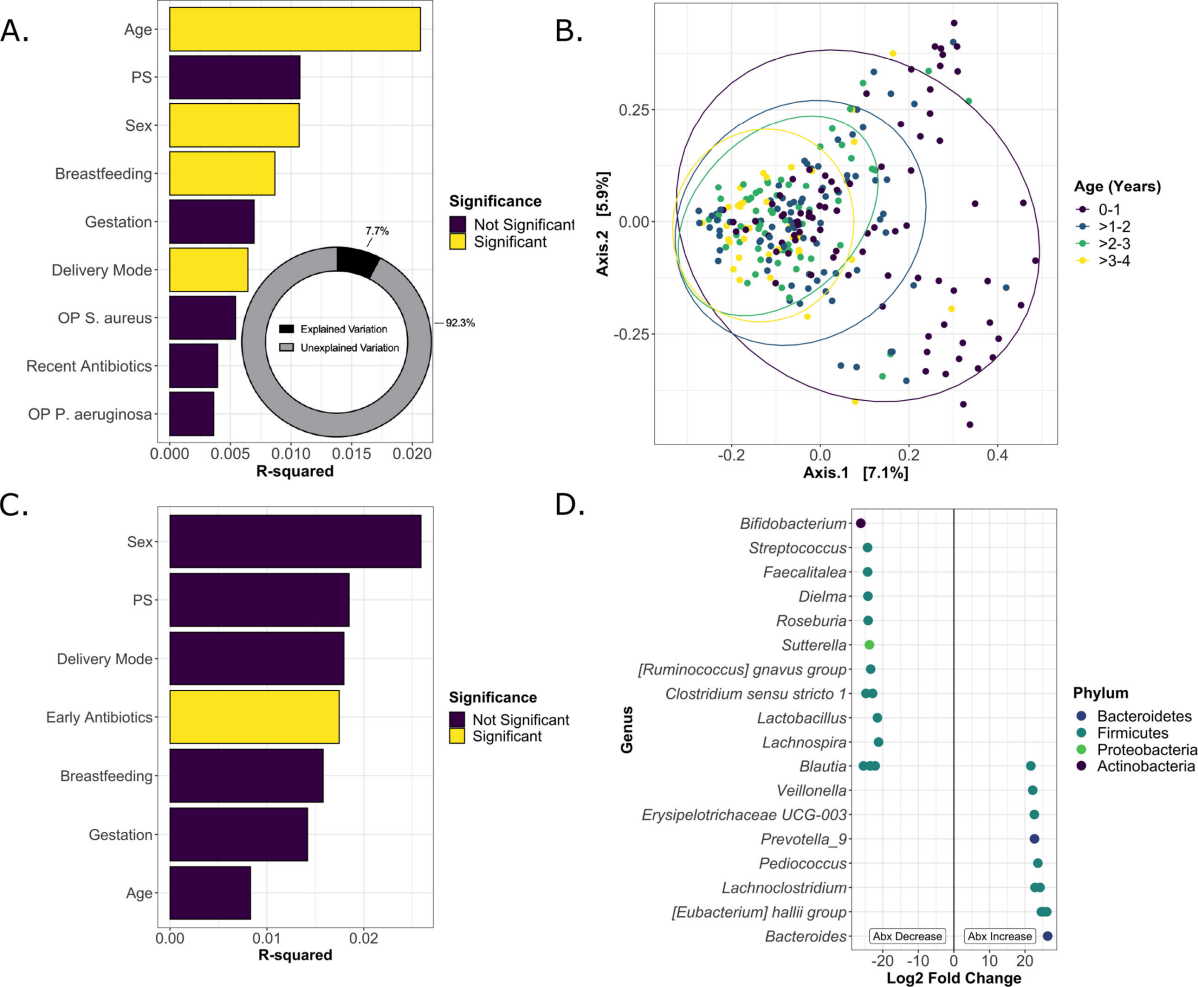
Beta diversity is associated with age, sex, breastfeeding, delivery mode, and antibiotics. (**A**) Bray–Curtis beta diversity was calculated for each sample. Significant differences in beta diversity were tested by PERMANOVA with demographic and exposure metadata included in the model, and R-squared values for all metadata included in the model were plotted with significant (*P* < 0.05) factors indicated. Total R-squared values were summed (inset). (**B**) Multidimensional scaling ordination of Bray–Curtis beta diversity was calculated for each sample. Significant differences in beta diversity were tested by PERMANOVA for each pair of 1 year age bins, with all samples included in the model to adjust for additional time points. Samples from cwCF <1 year of age were significantly different (*P* < 0.001) from all other time points. Samples from cwCF ages >1 to 2 were not significantly different from ages >2 to 3 (*P* = 0.25) but were significantly different from >3 to 4 (*P* = 0.01). Beta diversity for samples from cwCF ages >2 to 3 and >3 to 4 was not significantly different (*P* = 0.18). (**C**) R-squared values and significance were calculated as in (A) for samples from patients >2 years of age. Early antibiotic exposure, defined as exposure to any non-topical antibiotics before 6 months of age, was associated with significantly different beta diversity from 2 to 4 years of age. In all PERMANOVA tests, the strata function was used to adjust by individual subject for multiple sampling from the same cwCF. (**D**) Log_2_ fold change of taxa that were significantly altered in samples from subjects 2+ years of age who were and were not exposed to antibiotics at <6 months of age. A negative fold change represents a decrease in ASVs in subjects who were exposed to antibiotics early, while a positive fold change represents an increase in ASVs in subjects who were exposed to antibiotics early. Each dot represents a single ASV and is color coded by Phylum. Significance was determined by DESeq2 using a non-continuous model of samples. A single sample per subject was included to avoid multiple sampling; the oldest available sample was selected for each subject. Abx = Antibiotics.

The Bray–Curtis distance metric was used to test the influence of sex, pancreatic sufficiency, premature birth, delivery mode, breastfeeding, age, recent antibiotic exposure, and recent OP swab positivity on overall microbiome composition, and significance was tested by PERMANOVA (Fig. 2A). Age, sex, breastfeeding, and delivery mode all significantly impacted microbiome composition, with age having the largest *R*^2^ value. Interestingly, pancreatic sufficiency had a larger impact on microbiome beta diversity than breastfeeding or gestation despite being a statistically insignificant contributor. This finding is likely due to the low percentage of cwCF in our cohort who are pancreatic sufficient. However, it is notable that all combined exposures included in the model explained only 7.7% of the total variance in beta diversity, indicating that there is a large amount of variation between samples that are not due to any of the factors examined here (Fig. 2A).

We further analyzed the microbiome composition based on 1 year age bins and found that separation was strongest between samples <1 year of age relative to all other samples, which was significantly different from all other age groups (Fig. 2B). Furthermore, samples from the first year of life are highly dissimilar from each other and do not cluster tightly. Samples begin to cluster together more strongly after the first year of life, although samples from age 1 to 2 remain significantly different from other age groups. Beta diversity of samples collected from ages 2 to 3 and 3 to 4 years is not statistically significantly different from each other. Additional PERMANOVA tests between samples from the first 2 years of life grouped into 6 months age bins demonstrated a significant difference in composition between every pairwise comparison (0–6 months, 6–12 months, 12–18 months, and 18–24 months; *P* = 0.001) except for 12–18 months versus 18–24 months (*P* = 0.715). A shift in microbial composition is, therefore, apparent across approximately the first year and a half of life, with age being the largest driver of compositional changes.

We next wanted to determine whether early life exposures had detectable long-term impacts on the gut microbial composition of cwCF in this cohort. We used the Bray–Curtis distance metric to test the influence of sex, pancreatic sufficiency, premature birth, delivery mode, breastfeeding, age, recent antibiotic exposure, and early antibiotic exposure (defined as at least one non-topical antibiotic treatment <6 months of age) on overall microbiome composition from 2 to 4 years of age (Fig. 2C). Only early antibiotic exposure had a significant long-term impact on gut microbial community structure (*P* = 0.016). We next tested for significant differences in taxa at the ASV level in samples collected from 2 to 4 years of age from patients with and without early antibiotic exposures and found significant alterations of several ASVs (Fig. 2D). When long-term shifts in ASVs due to antibiotic exposure are compared with ASVs immediately altered by recent antibiotic exposure during the first 6 months of life (Fig. S2B), we see overlapping decreases in *Bifidobacterium, Streptococcus*, and *Blautia,* suggesting that these microbes are directly impacted by antibiotic exposure and may not recover. Early antibiotic exposure was significantly associated with long-term increases in several ASVs associated with CF lung disease, such as *Veillonella* and *Prevotella*, but a decrease in *Streptococcus*. Interestingly, *Bacteroides* ASVs are significantly decreased in the short term by antibiotic exposure, but there is a long-term increase in a single *Bacteroides* ASV with early antibiotic exposure. These data demonstrate that early antibiotic exposures may have long-term impacts on the gut microbial community.

### Large alterations in the microbiota occur in early life

**Fig 3:**
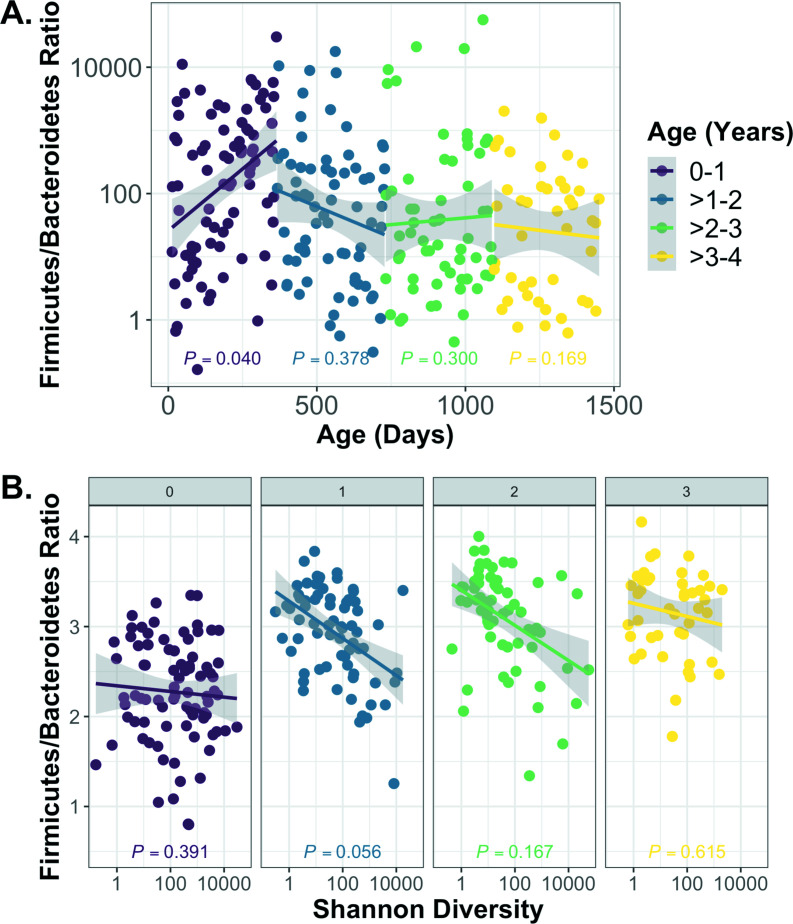
Firmicutes/Bacteroidetes ratios are age dependent. (**A**) For each sample, subject age in days is graphed versus the F/B ratio. A linear model was used to visualize correlations between age and ratios for each year of life. Statistical significance was tested by mixed linear model utilizing the R package nlme. Subject was set as the random variable to control for multiple sampling. F/B ratio was significantly correlated with age only in the first year of life but not after 1 year of age. (**B**) The Shannon Diversity Index is graphed versus the F/B ratio, and the correlation is displayed by linear model. Each panel is representative of 1 year age bins (0, 0–1 year; 1, 1–2 years, 2, 2–3 years, 3, 3–4 years). The F/B ratio was negatively associated with Shannon Diversity Index by mixed linear model for subjects aged 1–4 but not for subjects aged 0–1.

To understand the age-associated dynamics of the gut microbiome in cwCF, we first examined broad changes in relative abundance that occur over time at the phylum level. The ratio of Firmicutes/Bacteroidetes (F/B) has previously been associated with age and numerous health outcomes, including decreased F/B associated with inflammatory bowel disease and conflicting F/B ratios linked to obesity (36 - 39). In this cohort, the F/B ratio increased significantly over the first year of life but then dropped from ages 1 to 2 and plateaued over 2+ years (Fig. 3A; Fig. S3A). The F/B ratio was negatively associated with Shannon Diversity Index after the first year of life (Fig. 3B).

**Fig 4:**
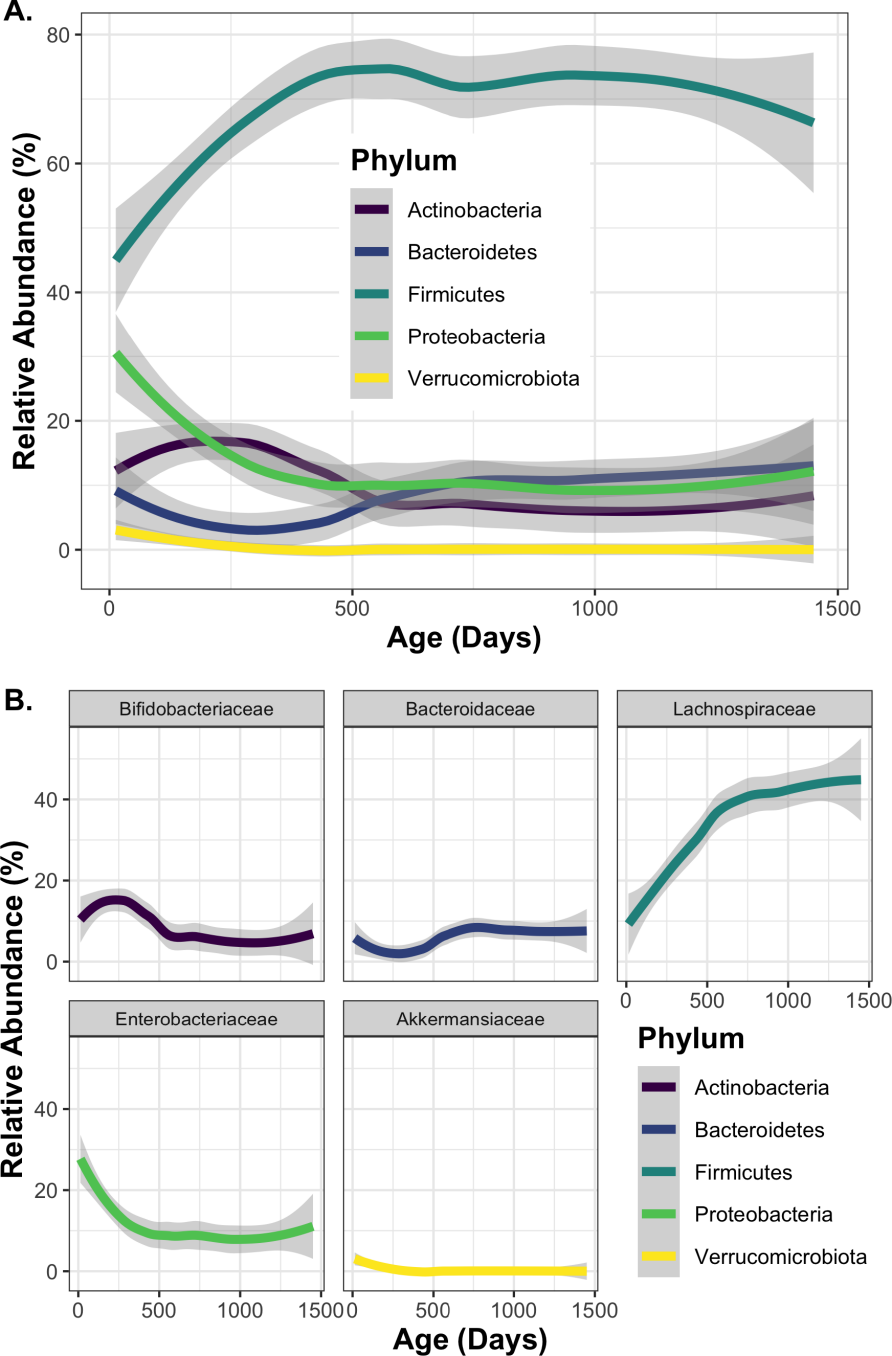
Microbial relative abundance changes with age. Age in days was graphed versus relative abundance of the indicated taxa for each sample, and a linear model was used to visualize overall changes in microbial relative abundance at the (A) phylum level and (B) family level over the first 4 years of life. The legend indicates the phylum to which each family belongs.

As cwCF age, major changes occur at the phylum level in early life and begin to stabilize after approximately 500 days (Fig. 4A). To further examine these changes, we compared average phylum-level relative abundances from all samples collected during four time windows: the first 6 months (182 days), between 6 months and 500 days, from 500 days to 2 years, and after 2 years (730 days) (Table 2). These time points were chosen to best reflect the early microbiome compared with samples collected within the stable period from 2 to 4 years of age. Firmicutes undergo the largest overall change, increasing from an average relative abundance of 51.5% to an average relative abundance of 71% (Fig. 4A; Table 2). Proteobacteria and Verrucomicrobiota relative abundances also change unidirectionally. Proteobacteria begin at 24.5% average relative abundance during the first 6 months of life and reduce to 11.3%, while Verrucomicrobiota decrease from an initial level of 1.5% down to less than 0.1%. Bacteroidetes and Actinobacteria stabilize at 10.5% and 7.1%, respectively, but trend in opposite directions over time, with Bacteroidetes showing a modest total increase after a reduction between the 6 months and 500 days age range.

**TABLE 2:** Relative abundance of major phyla for age-based subsets of samples^*a*^

Phylum	All samples	<6 mo	6 mo–500 days	500 days–2 yr	>2 yr
Firmicutes	66.3	51.5	71.0	72.3	71.0
Proteobacteria	14.2	24.5	12.3	10.3	11.3
Actinobacteria	10.7	14.9	14.1	9.34	7.10
Bacteroidetes	8.37	7.64	2.53	7.96	10.5
Verrucomicrobiota	0.36	1.50	0.06	0.07	0.05
Other	0.07	0.02	0.09	0.07	0.09

^a^ The average relative abundance of each taxa was first calculated by subject and then averaged across subjects for the given time period in order to control for multiple sampling per subject.

The phyla Actinobacteria, Bacteroidetes, Proteobacteria, and Verrucomicrobiota are dominated by single families: Bifidobacteriaceae, Bacteroidaceae, Enterobacteriaceae, and Akkermansiaceae, respectively, and phylum-level changes appear to be driven primarily by changes in these taxa (Fig. 4B; Fig. S3). The largest individual family changes in Firmicutes are seen for Lachnospiraceae, and changes in this family also reflect phylum-level changes (Fig. 4). However, Lachnospriraceae comprises <50% relative abundance of Firmicutes due to high diversity at the family level (Fig. S3). These results highlight how the highest-abundance taxa change with age for cwCF, and furthermore, the overall patterns appear to be driven by a small number of bacterial families, especially within the Actinobacteria, Bacteroidetes, Proteobacteria, and Verrucomicrobiota.

### Changes in the relative abundance of microbiota over time

**Fig 5:**
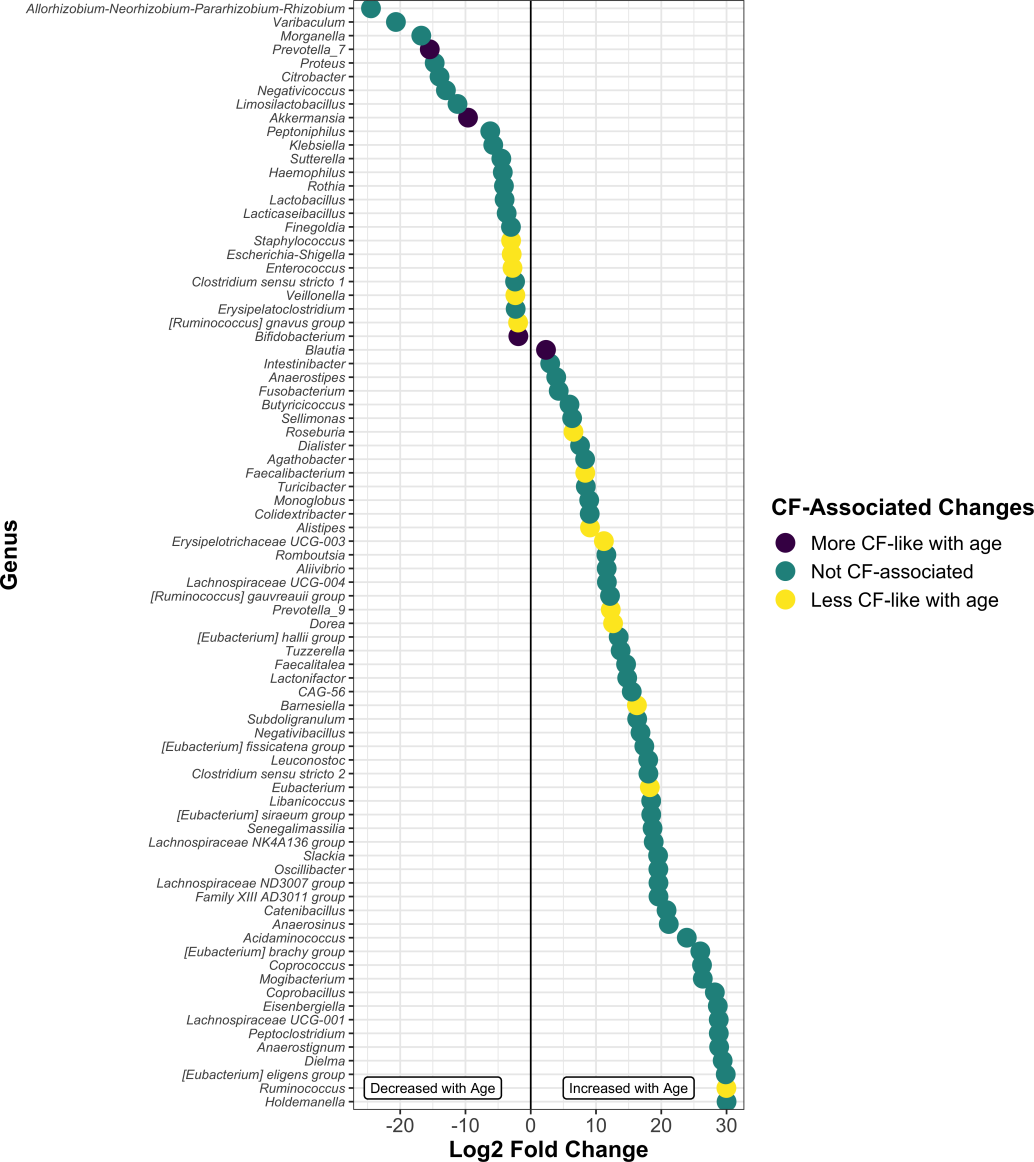
Significant alterations of abundance at the genus level occur with age. Log_2_ fold change of taxa that was significantly altered in samples from subjects 2+ years of age relative to subjects <6 months of age. Each dot represents a single genus and is color coded by known CF-associated changes from current literature (see main text for details). Significance was determined by DESeq2 using a non-continuous model of samples binned by age at sample collection. Subject was included as a design variable to control for multiple sampling.

We next determined the genera that changed significantly with age in our cohort. We compared a subset of samples grouped by age; samples <6 months and samples >2 years (Fig. 5; Table S2). Taxa that changed significantly with age were compared with taxa known to be altered in pwCF relative to non-CF cohorts to determine whether changes moved toward a “healthy-like” composition or a “CF-like” composition with age. Whether taxa are typically altered in pwCF relative to non-CF cohorts was determined using a summary of the CF gut microbiome literature that utilized results from twelve 16S and metagenomic studies of the CF gut microbiome (40). This analysis has been applied such that taxa that are known to be higher in pwCF are labeled here as “more CF-like” if the taxa increased with age and “less CF-like” if decreased with age. For taxa known to be decreased in pwCF, those that increase with age are labeled as “less CF-like,” and ones that decrease with age become “more CF-like.” For example, *E. coli* is known to be increased in the gut microbiota of cwCF (8, 28). We found that the *Escherichia/Shigella* group significantly decreased in relative abundance with increasing age, indicating that the relative abundance of *Escherichia/Shigella* shifts toward “healthy-like” with age (Fig. S4A). Notably, several taxa that are known to be decreased in pwCF, including butyrate-producers *Roseburia, Faecalibacterium, and Ruminococcus*, significantly increased with age in our cohort. The majority of taxa that changed significantly with age trended toward a “healthy-like” composition over time. These results support the idea that the gut microbiome becomes less dysbiotic with age for pwCF, as has been observed in previous studies (8, 9). However, the genus *Bacteroides,* which has reduced relative abundance in the gut microbiome of pwCF, does not significantly increase with age (Fig. S4B), in contrast to what has been reported in healthy cohorts. Additionally, four genera became more “CF-like” with age, including *Prevotella_7*, *Akkermansia*, *Bifidobacterium*, and *Blautia*. Of particular note is *Blautia*, which increases to >10% relative abundance in pwCF at >2 years of age (Fig. S4C) and is the only taxa identified to both increase in relative abundance and become more CF-like with age. Interestingly, *Blautia* has both positive and negative associations with health in non-CF populations (41 - 44) but has rarely been discussed in the CF gut microbiome literature.

### CF lung pathogens are detected in the gut

**Fig 6:**
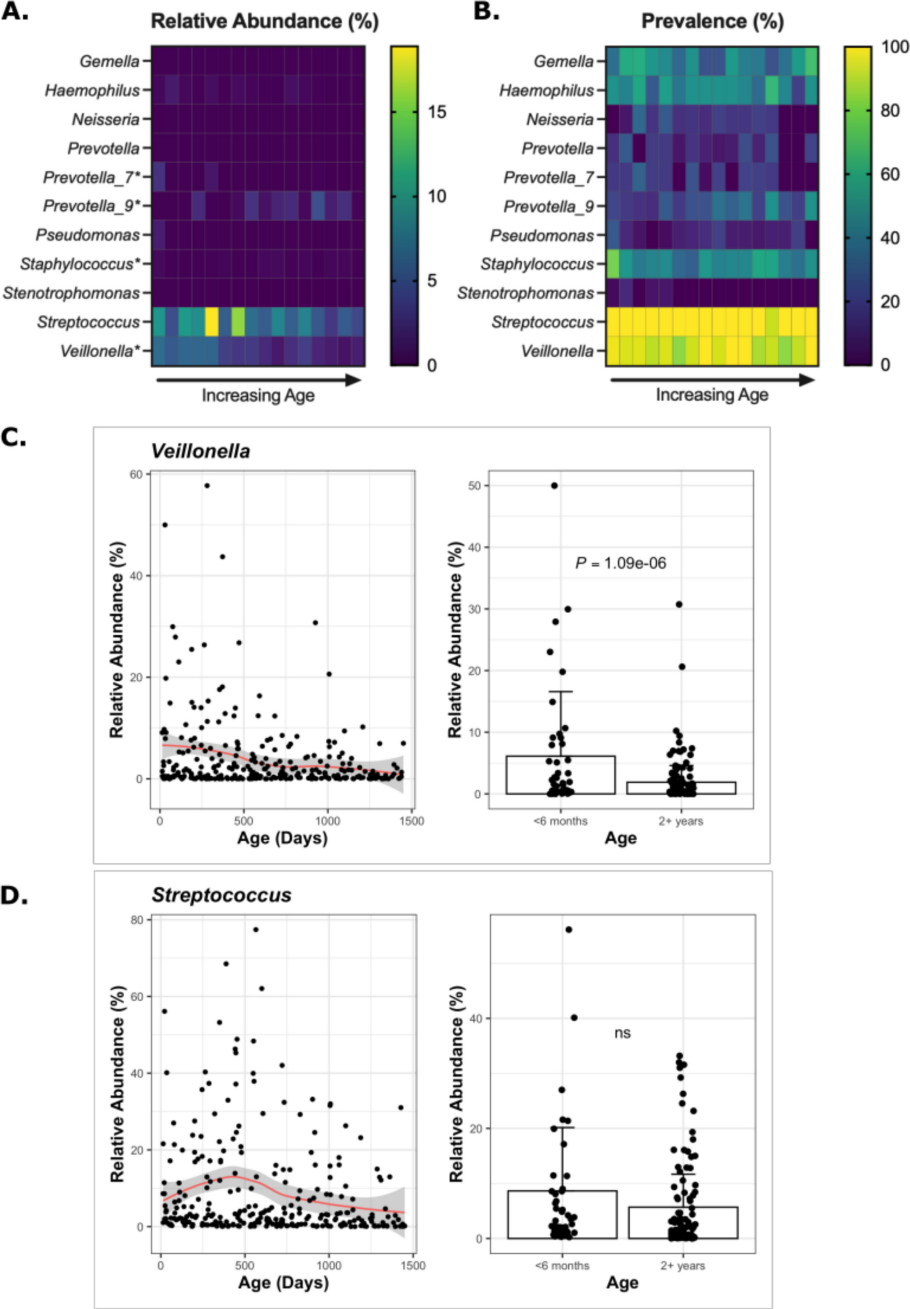
Age-related changes of genera associated with CF lung pathogenesis. (A) Relative abundance and (B) prevalence of each genus of interest in 3 month age bins. The displayed values were averaged first by subject to control for multiple sampling. (**C, D**) Changes over time of the relative abundance of *Veillonella* and *Streptococcus* are visualized by linear model (left), and the average relative abundance is displayed for all samples from subjects <6 months of age and 2+ years of age (right). Each dot represents a single sample. The displayed relative abundance was averaged first by subject to control for multiple sampling. ns, not significant (*P* = 1).

We examined each stool sample for both relative abundance and prevalence of opportunistic pathogens known to be important in CF, including *Gemella, Haemophilus, Neisseria, Prevotella, Pseudomonas, Staphylococcus, Stenotrophomonas, Streptococcus,* and *Veillonella* (Fig. 6A and B; Table S3). Several of these genera are not known gut residents, so may be present transiently and indicate oropharyngeal seeding of the gut. However, of greater interest is the possibility that some of these taxa may be seeding the lungs from the gut. Previous work by Madan et al. demonstrated that several lung pathogens are detectable in stool prior to detection in the lungs (7).

Four of the taxa examined here (*Haemophilus*, *Prevotella_7, Staphylococcus*, and *Veillonella*) had significantly higher relative abundance in the first 6 months of life compared with >2 years of age, while *Prevotella_9* significantly increased in the older cwCF (Fig. 5; Table S2A and B). *Streptococcus* and *Veillonella*, which are known to be gut resident microbes and have increased relative abundance in pwCF, had the highest overall relative abundance and prevalence in the gut of the known CF lung microbes (Fig. 6; Table S3). The relative abundance of *Veillonella*, but not *Streptococcus*, decreased significantly with age (Fig. 6C and D). Next most prevalent (~20%–50%) were *Prevotella, Haemophilus, Gemella,* and *Staphylococcus*. Of these, *Staphylococcus* and *Haemophilus* relative abundance decreased modestly but significantly with age. *Prevotella_7* and *Prevotella_9*, two unique ASVs within the Prevotellaceae family, also significantly changed with age, but *Prevotella_7* decreased with age while *Prevotella_9* increased with age. We observed lower rates of prevalence for *Stenotrophomonas, Pseudomonas,* and *Neisseria* at 1.8%, 9%, and 14%, respectively, and the relative abundances of these taxa do not change significantly with age. These data confirm that genera known to be important in CF lung disease are detected early and frequently in the gut microbiome of pwCF and could serve as a reservoir for seeding the lung.

### Early Crohn’s Dysbiosis Index predicts later outcomes

**Fig 7:**
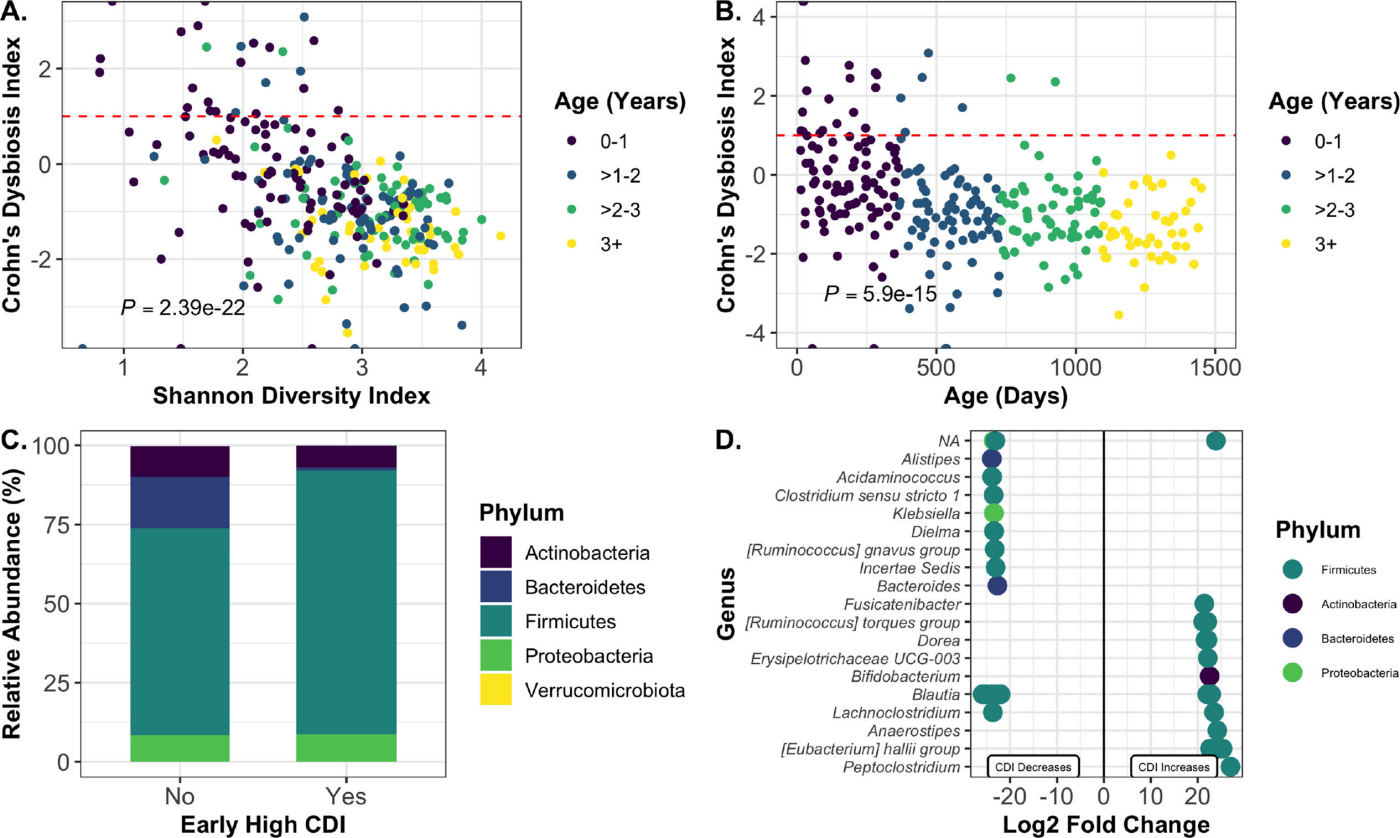
Early Crohn’s Dysbiosis Index is related to long-term changes in specific taxa. (**A**) Shannon Diversity Index and (B) age are plotted versus Crohn’s Dysbiosis Index for each sample. There was a significant inverse correlation between SDI and CDI as tested by mixed linear model with the R package nlme. Subject was set as the random variable to control for multiple sampling. (**C, D**) Samples from subjects age 2 and above were grouped by whether the subject had an earlier sample with CDI >0.5 (“yes,” *n* = 12) or whether all earlier samples had a CDI <0.5 (“no,” *n* = 11). The latest collected sample from each subject was analyzed. (**C**) Phylum-level relative abundances of samples with or without early high CDI. (**D**) Log_2_ fold changes of ASVs that were significantly different in the early high dysbiosis group versus the early low dysbiosis group. Significance was assessed with DESeq2.

Crohn’s disease is an intestinal inflammatory disorder with immunological and physiological symptoms overlapping with CF. The CDI has previously been applied to gut 16S rRNA gene amplicon sequencing data from cohorts of pwCF as a measure of gut dysbiosis and was positively correlated with higher levels of the inflammatory marker fecal calprotectin (12). We applied the CDI to our samples and found that only 25 of 281 samples exceeded the “severe Crohn’s” metric of CD >1 (Fig. 7A; Table S1).

Interestingly, when looking at the trends of cwCF individually, there did not appear to be a subset of subjects with a consistently high CDI (Fig. S5A). Instead, CDI was significantly negatively correlated with age, and most high CDI scores occurred during the first year of life, although not all cwCF had high scores at this age. CDI was also significantly negatively correlated with SDI; this is unsurprising given that microbiome diversity and age are highly intertwined (Fig. 7B). However, the only other study to apply the CDI in pwCF did not find a correlation between SDI and CDI (12); these differences are likely due to age differences between the cohorts.

To determine whether a high CDI early in life could potentially shape later microbiome structure, we took samples from >2 years for all subjects and classified them by whether or not the subject had at least one sample with a CDI >0.5 during the first 2 years of life. Samples were excluded from the analysis if the subject did not have at least one sample before and after age 2. A total of 23 cwCF in this study had samples that met these parameters, and a single sample from the latest collected time point was selected for each of these individuals. Samples were classified as having either “high” (>0.5) or “low” (<0.5) early CDI. Shannon diversity was not significantly different between the two groups, although it did trend higher in the early CDI group (Fig. S5B). However, the gender distribution of the two groups was significantly different by chi-squared test (*P* = 0.026), with more males in the early high CDI group (Fig. S5C). While this is a confounding factor that cannot be controlled for, it points to a potential influence of gender on early gut microbiome dysbiosis that is also reflected in overall sex-based differences in beta diversity (Fig. 2A). There were no significant differences in breastfeeding (*P* = 0.4128), delivery mode (*P* = 0.4058), gestation (*P* = 0.4128), pancreatic sufficiency (*P* = 0.2224), or early antibiotic exposure (*P* =0.2264) between the two CDI groups by chi-squared test (Fig. S5D–H). However, the rate of early antibiotic exposures did trend higher in the early high CDI group. Interestingly, the total number of antibiotic exposures was also not significantly different between the early high CDI group and children without early CDI at any time within the first 4 years of life, suggesting that the differences between the groups are not driven by total antibiotic exposures (Fig. S6A) but may be related to early antibiotic exposure.

Analysis of taxa at the phylum level demonstrated that the relative abundance of Bacteroidetes was lower for subjects who had a high early CDI (Fig. 7C; Table S4). We used DESeq2 to determine significantly different ASVs between these groups (Fig. 7D; Table S4). The overall decrease in Bacteroidetes at the phylum level is driven by a significant decrease in *Bacteroides*. However, not all of the changes were negative, as this group also saw a significant increase in the anti-inflammatory genus *Anaerostipes*. Interestingly, *Bifidobacterium* was increased in the early high CDI group. While *Bifidobacterium* is generally considered to be a beneficial microbe, it is also a marker of the early infant gut and thus may be an indication that the cwCF with early high CDI have a less mature gut microbiome composition than children without early high CDI. These findings highlight the fact that early microbiome disturbances may shape later outcomes, particularly for the genera *Bacteroides*.

Next, we tested whether early high CDI might influence colonization of the upper respiratory tract in cwCF. To explore this idea, we examined the average *S. aureus* and *P. aeruginosa* positivity of nasal swabs from cwCF with and without early high CDI throughout the first 4 years of life. We found that colonization with *S. aureus* trended higher over the first year of life for children with early high CDI, but no significant differences in colonization with either pathogen were detected between groups (Fig. S6B and C).

### Summary

We have performed a longitudinal analysis of microbiome samples from a cohort of 39 cwCF from birth through 4 years of age. For CF, which is a rare disease, this is a relatively large cohort with frequent longitudinal sampling and analysis of 281 total samples. We have demonstrated that the microbiome of cwCF follows some of the expected developmental patterns observed for infants and young children without CF, including beta diversity separation by age and increasing alpha diversity with age, although diversity plateaus ~2 years of age for this CF cohort. An advantage of our cohort is the ability to leverage matched clinical data with the microbiome data. We have done that here with subject sex, pancreatic sufficiency, breastfeeding, gestational age, delivery mode, antibiotic exposure, and oropharyngeal swab data. We observed significant negative impacts of preterm birth and recent antibiotic exposure on alpha diversity. Age, sex, breastfeeding, and delivery mode significantly impacted beta diversity, with age being the strongest driver of dissimilarity. When examining long-term impacts of exposures, early antibiotic treatment has a significant impact on the 2–4-year microbiota structure.

Our findings have confirmed that many of the taxa known to be specifically increased or decreased in pwCF have the most extreme dysbiosis in early life and tend to move toward more typical abundances as cwCF age (8, 9). A few microbes differ from this pattern, including *Akkermansia*, which is higher early in life in this cohort and is known to be decreased in pwCF. Furthermore, we note that *Bacteroides*, which is reduced in pwCF and may play an important role in immune programming, does not significantly increase with age. We have also confirmed the presence of many CF lung microbes in the gut of cwCF, highlighting the possibility of the lung being seeded directly from the gut early in life, consistent with previous findings (7). Interestingly, early antibiotic treatment is associated with a longer-term increase in some CF lung-associated microbes. These increases may be due to a direct effect of antibiotics on microbial community composition but are likely a more complex reflection of how the need for early antibiotic intervention interacts with disease progression and the gut–lung axis in CF.

Finally, we have applied the CDI to our cohort in a novel way. To date, the CDI has only been applied once in pwCF and not with a longitudinal dataset (12). The CDI has previously been associated with calprotectin, a marker of intestinal inflammation, in both persons with Crohn’s disease and pwCF (12, 34). These two diseases share many overlapping intestinal symptoms, and some tools from Crohn’s clinical diagnostics, such as the use of calprotectin as a marker of intestinal inflammation (45), have been used in recent CF studies (11, 20, 22, 28, 46, 47). The CDI may, therefore, be another useful tool that can be employed to better understand the dynamics of the intestinal microbiome in pwCF and its relationship with clinical outcomes. Interestingly, we found that early high CDI appears to be associated with later microbiome structure (in years 2–4), particularly for *Bacteroides*. Previous work from our group and others demonstrated that *Bacteroides* is reduced in cwCF <1 year of age (4, 9, 11). This current work extends that finding to later childhood and demonstrates that early perturbations can have long-term effects on *Bacteroides* relative abundance. Interestingly, children with early high CDI scores do not have significantly more total or early antibiotic exposure, indicating that these gut microbiome alterations are not driven solely by antibiotic interventions.

With this analysis, we are able to better understand the development of the CF intestinal microbiome throughout early childhood. However, one caveat to this work is that, as with all clinical cohorts, our findings are observational in nature, and causation cannot be attributed to any one or combination of factors. Furthermore, while our cohort is quite large for a CF study, the high level of heterogeneity within the patient population and treatment regimens prevented finer-grained analysis of some exposures, such as antibiotics by class or administration route. Larger cohorts and/or longer longitudinal studies may be useful for better understanding the influence of antibiotics, dietary interventions, and CF-specific medications. Future work within this cohort will focus on understanding how early alterations in the gut microbiome impact later lung health outcomes as patients within our cohort age. This analysis will help us to better understand how CF influences the microbiome and how microbiome development impacts the gut–lung axis.

## Data Availability

All sequence reads can be found in GenBank Sequence Read Archive with sequences found under accession number  SRP014429 and BioProject ID 170783 under accession number PRJNA170783

## SUPPLEMENTAL MATERIAL

Fig S1 msphere.00046-23-s0001.pdf Shannon Diversity Index changes significantly with age, gestation, and antibiotic exposure.

Fig S2 msphere.00046-23-s0002.pdf Antibiotic exposure.

Fig S3 msphere.00046-23-s0003.pdf Relative abundances of taxa over time and at the family level.

Fig S4 msphere.00046-23-s0004.pdf Alterations of taxa with age.

Fig S5 msphere.00046-23-s0005.pdf Crohn’s Dysbiosis Index.

Fig S6 msphere.00046-23-s0006.pdf Antibiotic and upper respiratory tract colonization are not associated with early high CDI.

TABLE S1 msphere.00046-23-s0007.xlsx Sample data, Shannon Diversity Index, and Crohn’s Dysbiosis Index.

TABLE S2 msphere.00046-23-s0008.xlsx Significantly altered taxa and all amplicon sequence variants.

TABLE S3 msphere.00046-23-s0009.xlsx Relative abundances of CF lung pathogens detected in stool samples.

TABLE S4 msphere.00046-23-s0010.xlsx Influence of early CDI on late microbiota composition.

ASM does not own the copyrights to Supplemental Material that may be linked to, or accessed through, an article. The authors have granted ASM a non-exclusive, world-wide license to publish the Supplemental Material files. Please contact the corresponding author directly for reuse.
