# Supplementary material for: Development of the intestinal microbiome in cystic fibrosis in early life
Source: mSphere. 2023 Jul 5;8(4):e00046-23. doi: 10.1128/msphere.00046-23 (PMC10449510; doi:10.1128/msphere.00046-23)
Supplement: Fig S1 — Shannon Diversity Index changes significantly with age, gestation, and antibiotic exposure. [file msphere.00046-23-s0001.pdf]

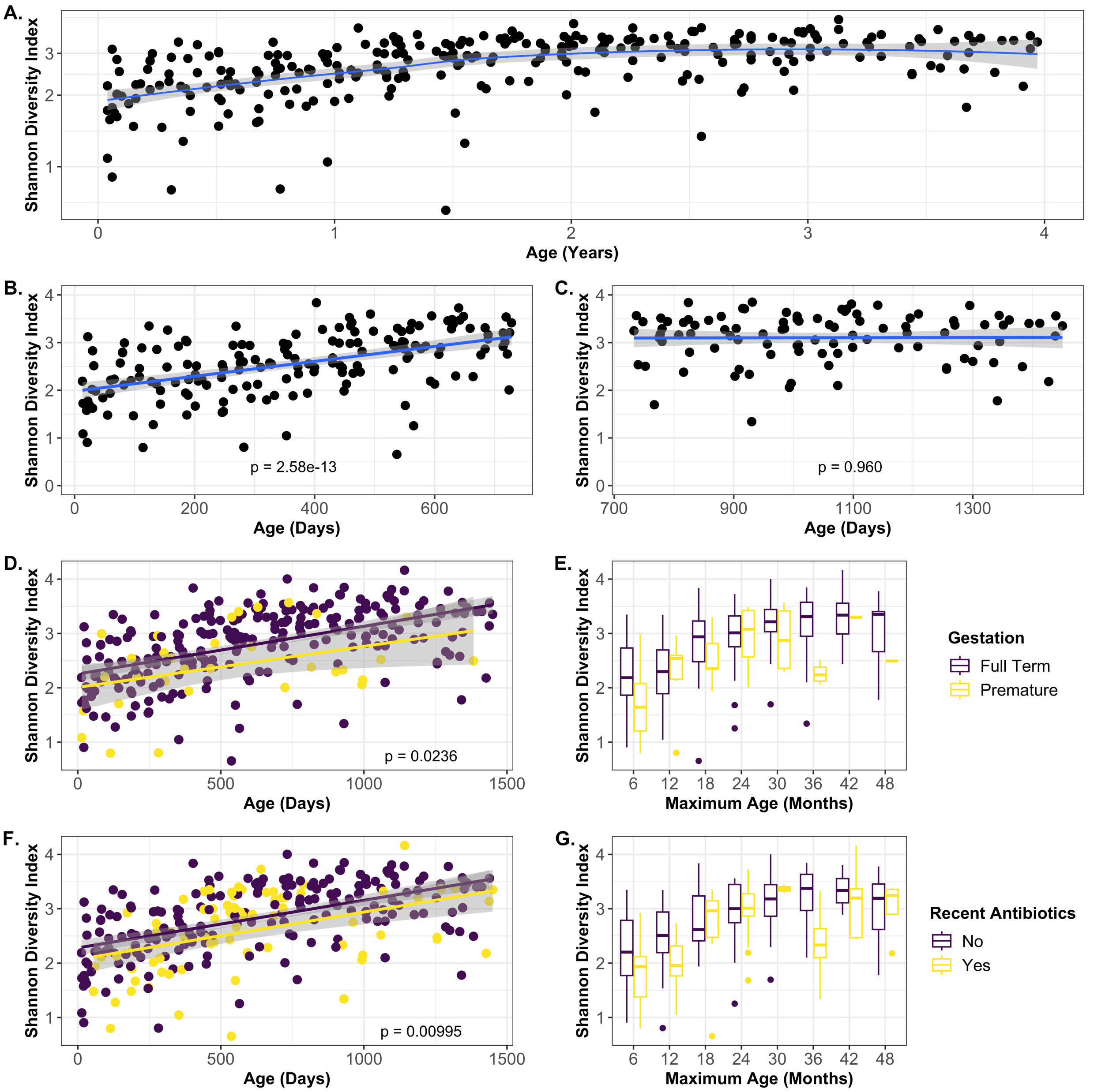

Figure S1. Shannon Diversity Index changes significantly with age, gestation, and antibiotic exposure. A) Age versus Shannon Diversity Index of samples is visualized by individual data points and a LOESS smooth line generated in R by the geom_smooth() function. A linear mixed effects model from the R package nlme was used to test whether SDI changed significantly with B-C) age in days, D-E) age at gestation, or F-G) antibiotic exposure within the previous 60 days. Subject was set as the random variable to control for multiple sampling. P-values are displayed on the graph. B-C) Figure displays mixed linear regression for samples in 2-year age bins from

B) 0-2 years and C) >2-4 years. E, G) Boxplots of Shannon Diversity Index binned in 6-month age increments.
