# Supplementary material for: Development of the intestinal microbiome in cystic fibrosis in early life
Source: mSphere. 2023 Jul 5;8(4):e00046-23. doi: 10.1128/msphere.00046-23 (PMC10449510; doi:10.1128/msphere.00046-23)
Supplement: Fig S2 — Antibiotic exposure. [file msphere.00046-23-s0002.pdf]

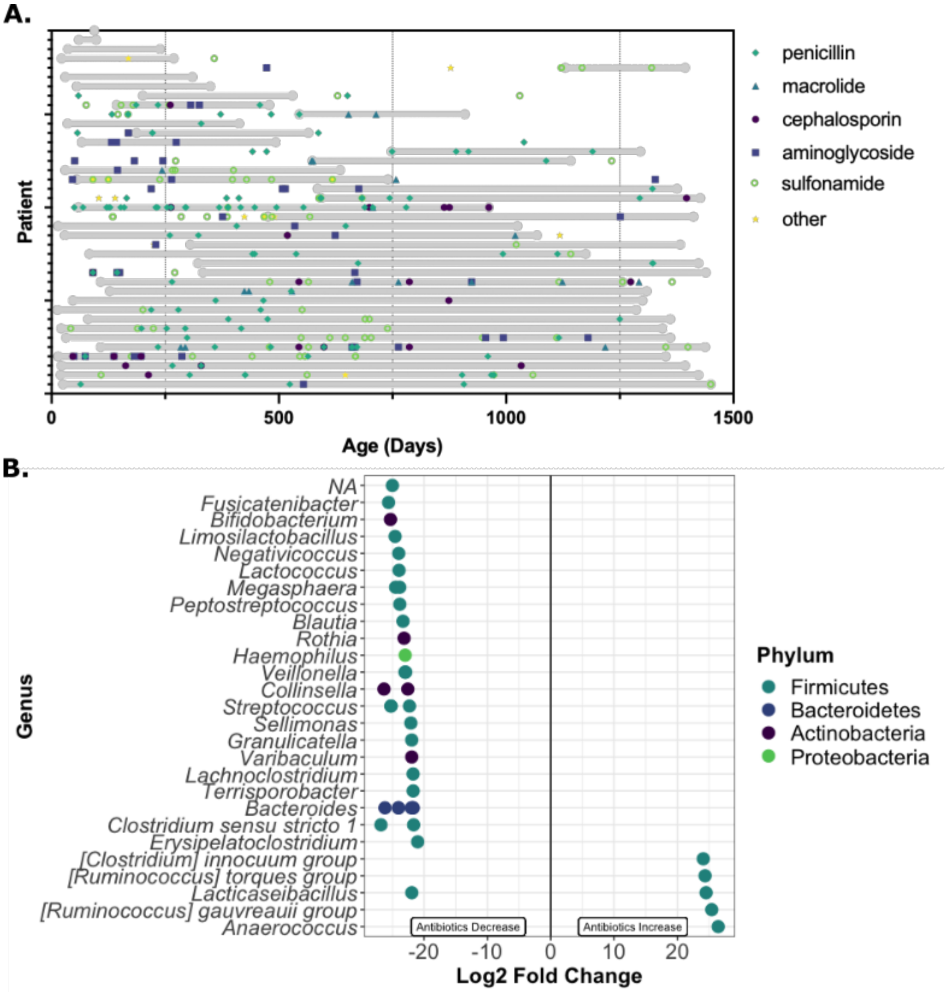

Figure S2. Antibiotic exposure. A) Summary of antibiotic exposure for all subjects in this study. Shown are the antibiotics for each subject in this cohort, illustrating the complexity and variability of this exposure over the first years of life. Each point represents a single antibiotic and the class of antibiotic is indicated by the shape and color of the symbol (see legend). Each gray bar represents the peroid of time across which stool samples were collected for each subject. Antibiotic information is available outside the window of stool sample collection for some but not all subjects. B) Log_2 fold change of taxa that were significantly altered in samples from subjects <6 months of age who were and were not exposed to antibiotics within the previous 60 days. A negative fold change represents a decrease in ASVs in subjects who were exposed to

antibiotics, while a positive fold change represents an increase in ASVs in subjects who were exposed to antibiotics. Each dot represents a single ASV and is color-coded by Phylum. Significance was determined by DESeq2 using a non-continuous model of samples and subject was included as a design variable to control for multiple sampling.
