# Supplementary material for: Development of the intestinal microbiome in cystic fibrosis in early life
Source: mSphere. 2023 Jul 5;8(4):e00046-23. doi: 10.1128/msphere.00046-23 (PMC10449510; doi:10.1128/msphere.00046-23)
Supplement: Fig S3 — Relative abundances of taxa over time and at the family level. [file msphere.00046-23-s0003.pdf]

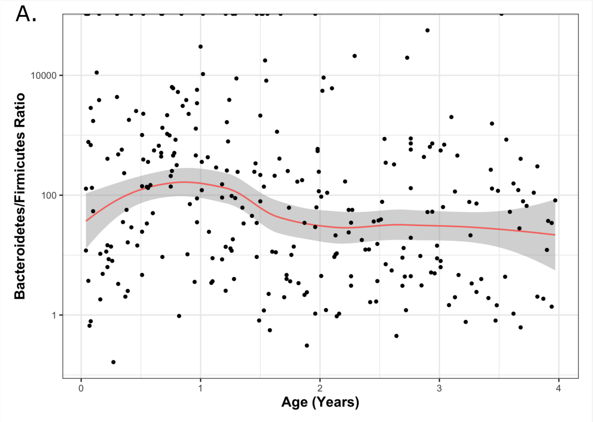

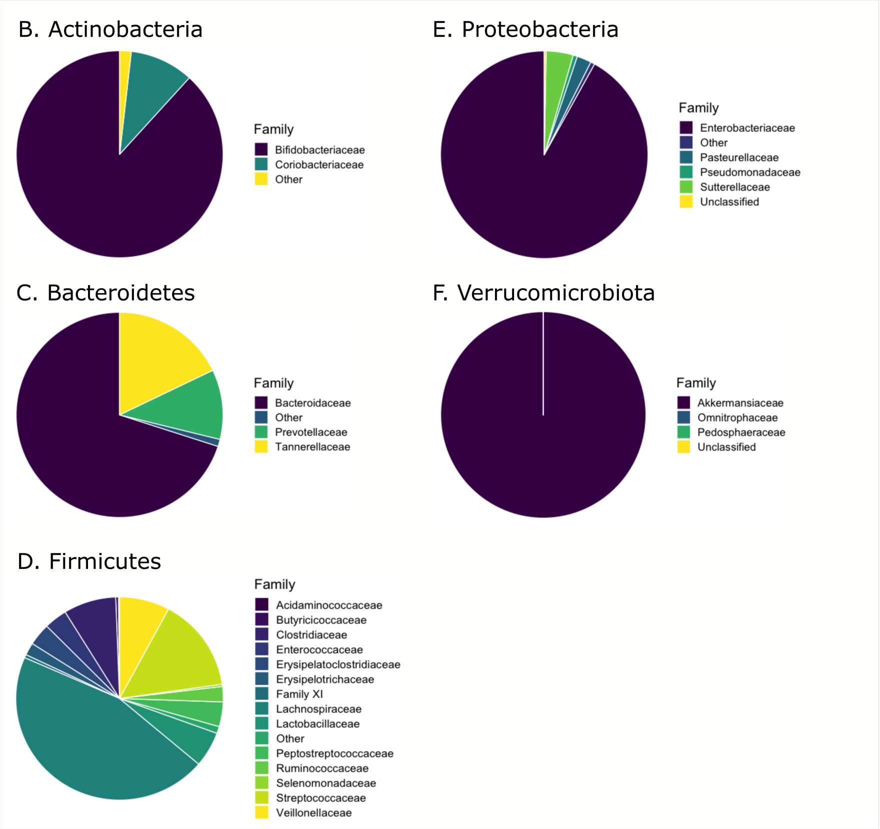

Figure S3. Relative abundances of taxa over time and at the family level. A) Age versus Bacteroidetes/Firmicutes ratio of samples is visualized by individual data points and a LOESS smooth line generated in R by the geom_smooth() function. B-F) Relative abundances of taxa at the family level are displayed for each phylum. Taxa with relative abundances within the phylum <10% are classified as “Other”.
