# Supplementary material for: Development of the intestinal microbiome in cystic fibrosis in early life
Source: mSphere. 2023 Jul 5;8(4):e00046-23. doi: 10.1128/msphere.00046-23 (PMC10449510; doi:10.1128/msphere.00046-23)
Supplement: Fig S4 — Alterations of taxa with age. [file msphere.00046-23-s0004.pdf]

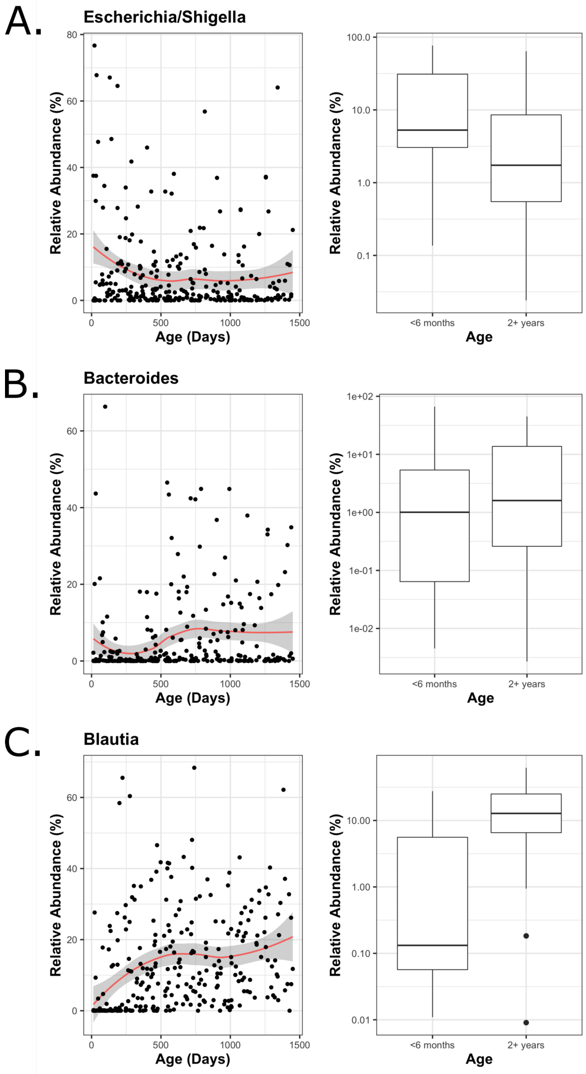

Figure S4. Alterations of taxa with age. For each panel, age versus genera-level relative abundances of samples is visualized by linear model (left) and the average relative abundances for samples from cwCF <6 months of age and cwCF 2+ years of age (right) are plotted for A) *Escherichia/Shigella* (p=5.13e-6), B) *Bacteroides* (p = 0.95) and C) *Blautia* (p=3.33e-5). Significance was tested between samples from cwCF <6months of age versus 2+ years of age (right) by DESeq2 (Table S2). Patient was included as a design variable to control for multiple sampling.
