# Supplementary material for: Development of the intestinal microbiome in cystic fibrosis in early life
Source: mSphere. 2023 Jul 5;8(4):e00046-23. doi: 10.1128/msphere.00046-23 (PMC10449510; doi:10.1128/msphere.00046-23)
Supplement: Fig S5 — Crohn’s Dysbiosis Index. [file msphere.00046-23-s0005.pdf]

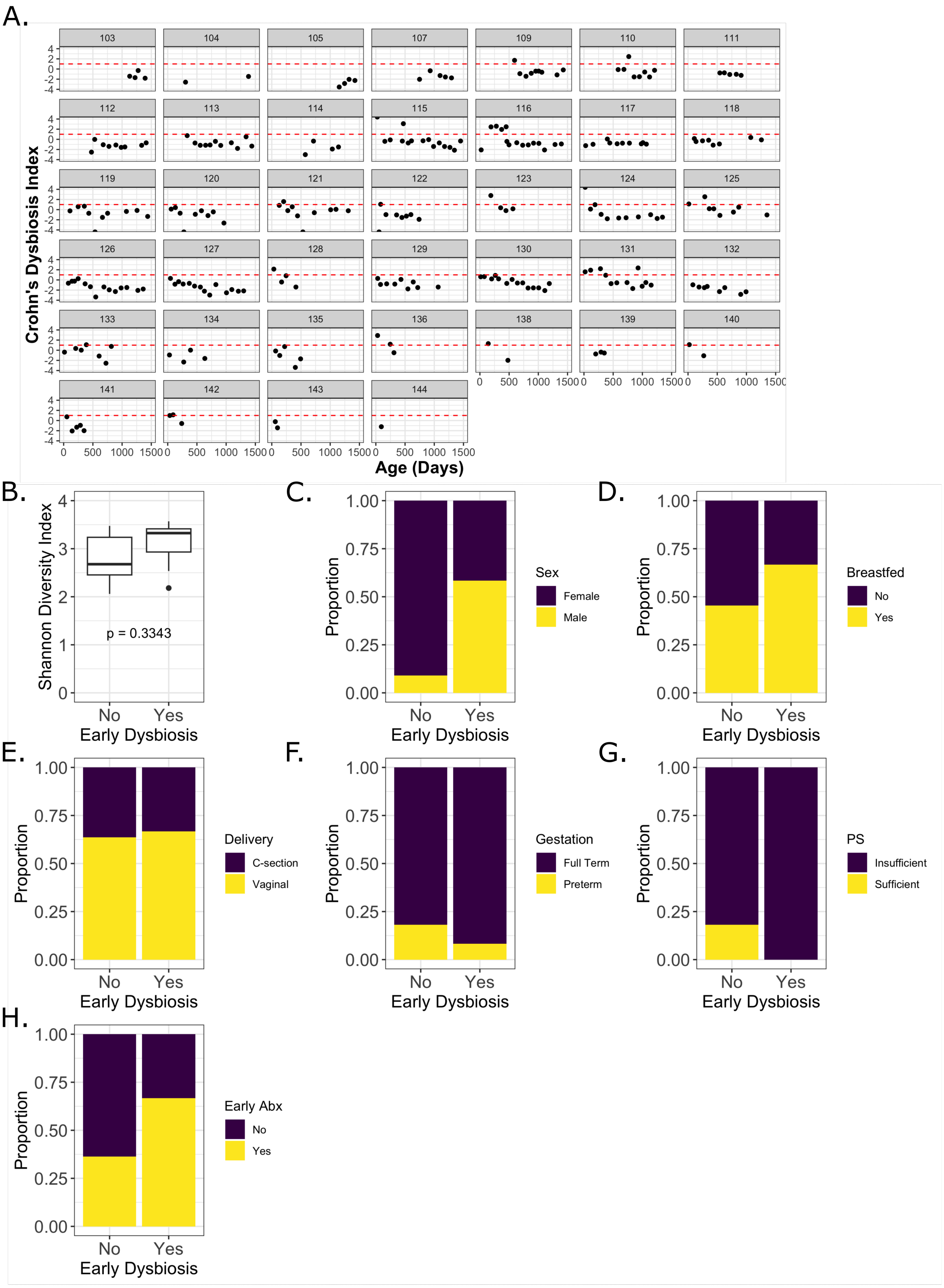
A.
103
104
105
107
109
110
111
112
113
114
115
116
117
118
119
120
121
122
123
124
125
126
127
128
129
130
131
132
133
134
135
136
138
139
140
141
142
143
144
Crohn's Dysbiosis Index
Age (Days)
B.
Shannon Diversity Index
p = 0.3343
No
Yes
Early Dysbiosis
C.
Proportion
Sex
Female
Male
Early Dysbiosis
D.
Proportion
Breastfed
No
Yes
Early Dysbiosis
E.
Proportion
Delivery
C-section
Vaginal
Early Dysbiosis
F.
Proportion
Gestation
Full Term
Preterm
Early Dysbiosis
G.
Proportion
PS
Insufficient
Sufficient
Early Dysbiosis
H.
Proportion
Early Abx
No
Yes
Early Dysbiosis

Figure S5. Crohn's Dysbiosis Index. A) Age versus Crohn's Dysbiosis Index for each sample displayed by individual subject. The dashed red line is set at 1, the cutoff for severe Crohn's in the original publication. B) Shannon Diversity Index, C) sex, D) breastfeeding, E) delivery mode, F) gestation, G) pancreatic sufficiency (PS), and H) early antibiotic exposure (prior to 6 months of age) distribution for patients with ('Yes', n=12) or without ('No', n=11) an early high dysbiosis score. Differences in Shannon Diversity Index are not significant by student's t-test ($p=0.33$). Differences in sex distribution are significant by chi-squared test ($p=0.02599$). Breastfeeding ($p = 0.4128$), delivery mode ($p= 0.4058$), gestation ($p = 0.4128$), pancreatic sufficiency ($p = 0.2224$), and early antibiotic exposure ($p = 0.2264$) were not significant by chi-squared test.
