# Supplementary material for: Development of the intestinal microbiome in cystic fibrosis in early life
Source: mSphere. 2023 Jul 5;8(4):e00046-23. doi: 10.1128/msphere.00046-23 (PMC10449510; doi:10.1128/msphere.00046-23)
Supplement: Fig S6 — Antibiotic and upper respiratory tract colonization are not associated with early high CDI. [file msphere.00046-23-s0006.pdf]

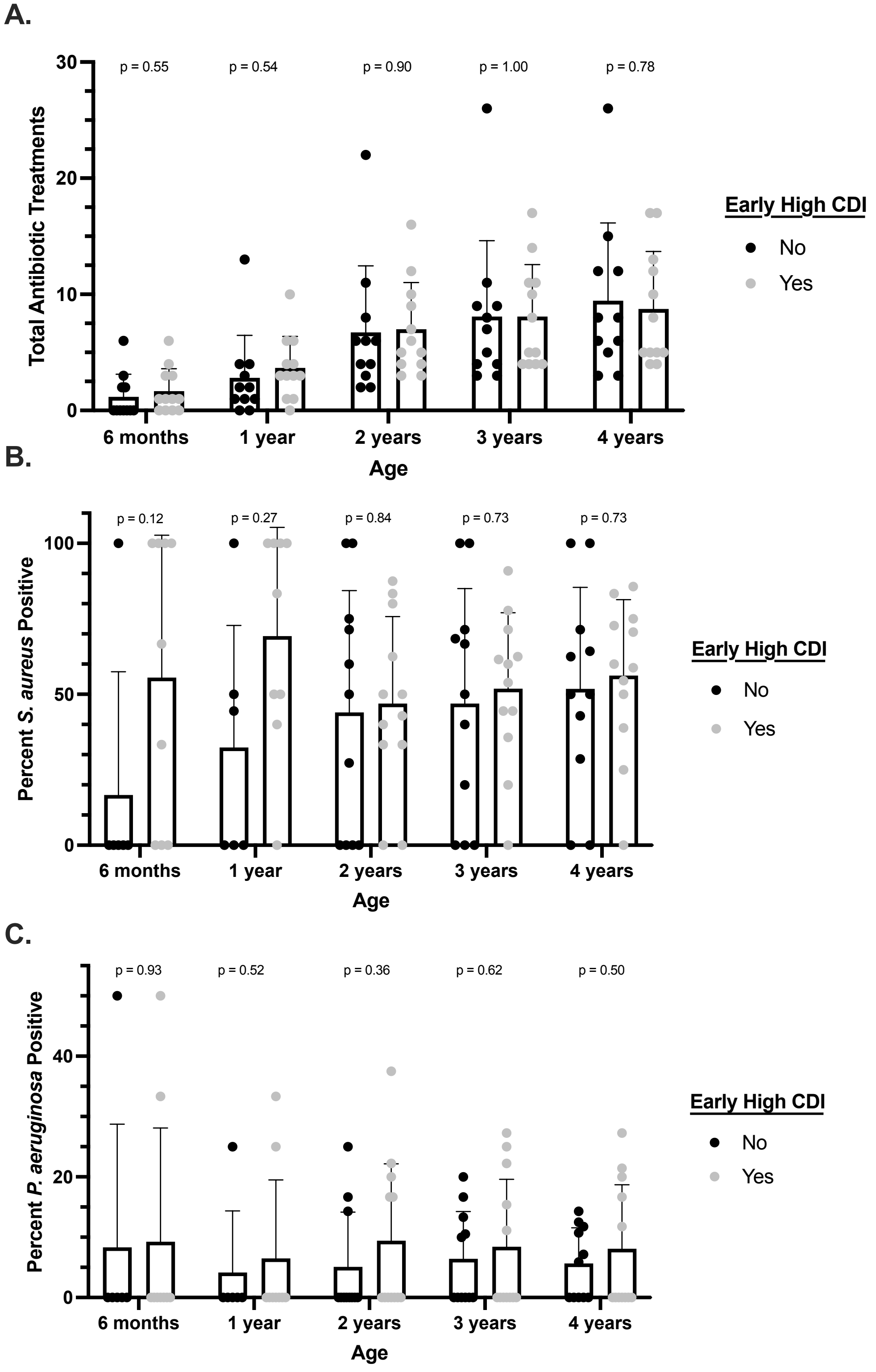

A.
Total Antibiotic Treatments
p = 0.55
p = 0.54
p = 0.90
p = 1.00
p = 0.78
30
20
10
0
6 months
1 year
2 years
3 years
4 years
Age
Early High CDI
No
Yes
B.
Percent S. aureus Positive
p = 0.12
p = 0.27
p = 0.84
p = 0.73
p = 0.73
100
50
0
6 months
1 year
2 years
3 years
4 years
Age
Early High CDI
No
Yes
C.
Percent P. aeruginosa Positive
p = 0.93
p = 0.52
p = 0.36
p = 0.62
p = 0.50
40
20
0
6 months
1 year
2 years
3 years
4 years
Age
Early High CDI
No
Yes

Figure S6. Antibiotic and upper respiratory tract colonization are not associated with early high CDI. A) The total number of antibiotic treatments for each subject was summed from birth through the indicated age. Significant differences were tested by paired t-test for each time point for cwCF with and without early high CDI. All comparisons were not significant, and p-value is indicated in the figure. The total percentage of upper respiratory tract cultures that were positive for each patient for B) *S. aureus* and C) *P. aeruginosa* were calculated from birth through the indicated age. Significant differences were tested by paired t-test for each time point for cwCF with and without early high CDI. All comparisons were not significant, and p-values are indicated on the figure.
